# Supplementary material for: The Role of Workplace on Work Participation and Sick Leave after a Terrorist Attack: A Qualitative Study
Source: Int J Environ Res Public Health. 2021 Feb 17;18(4):1920. doi: 10.3390/ijerph18041920 (PMC7921948; doi:10.3390/ijerph18041920)
Supplement: Supplementary file 1 [file ijerph-18-01920-s001.pdf]

Supplementary Data

Analysis of the data material

All interviews were imported in their entirety to the software package NVivo as transcribed text files. Using NVivo facilitated the work of organizing data, reflecting during the work, exploring the data material and integrating the results. The analysis followed a six-step process. In the first step we read and re-read each of the interviews. We made notes in NVivo on tentative ideas about coding and underlying themes that we compared. The purpose was to become familiar with the material, identify repetitive patterns, how these were communicated and to what extent they were important to the participant.

The purpose was to become familiar with the material, identify repetitive patterns, how these were communicated and to what extent they were important to the participant.

Hensikten var å bli kjent med materialet, identifisere repeterende mønstre, hvordan disse ble kommunisert og i hvilken grad de var viktige for deltakeren.

The purpose was to get acquainted with the material, identify recurring patterns, how these were conveyed and the extent to which they were important for the participant.

Hensikten var å bli kjent med materialet, identifisere gjentatte mønstre, hvordan disse ble formidlet og i hvilken grad de var viktige for deltakeren.

Kan ikke laste inn fullstendige resultater

Prøv på nytt

Prøver på nytt …Prøver på nytt …

In the second step of the analysis, we went systematically through the transcript of every interview and identified by codes everything that looked potentially interesting from the perspective of the participant. Each code described an idea, feeling or experience expressed in the text. These were added to the systematic overview in NVivo. An example of a code was "having a leader without expectation that I would perform". The coding was empirically driven, in that codes and themes were based on the data material, rather than existing theories or hypotheses. In order to meet criticism of thematic analysis that quotations are often taken out of context, longer rather than short quotations were chosen. In this step, emphasis was placed on not excluding anything that could be of potential relevance to the participant. The coding of all the interviews yielded 149 codes.

In the third step, we sorted codes and associated text extracts under potential themes that expressed a broader perspective than each code. We identified the themes in NVivo by evaluating each code and associated text. Similar codes were placed under the same theme. This organization of codes yielded 105 potential themes.

In the fourth step, we reviewed the potential themes. We used functions such as *comparison diagram*, *hierarchy chart* and *mind map* in NVivo to create thematic maps, and we formed sub-themes to create structure in cases where there were large and complex themes. Themes that turned out to contain the same text extracts were merged. Themes that seemed less relevant to the participants were removed. For example, several of the participants reported that they had been followed up by the occupational health service, without mentioning it as something that was important for their sick leave or work participation. Status after the review was 10 themes and 101 sub-themes (Table S1).

In the fifth step, each theme was redefined and specified according to the entirety of the data material and relevance for the research questions. It turned out that that several sub-themes ran across the main themes. We therefore ended up renaming the themes and reorganizing the sub-themes accordingly. The characteristics of a manager who stimulated employees' capacity to work were gathered under the final theme *supportive management*. Autonomy and flexibility were moved from the theme *work* (Table S1) to *supportive management*, as the data material revealed the importance of a manager who facilitated the two characteristics among their employees.

For the theme *individual* (Table S1), factors were redefined under the final theme *working as a coping strategy*, which also included the expectation of continuing to work in the same way as before the bomb explosion (moved from *social conditions*), and mastery beliefs and encouragement to be present (moved from *manager*). Although these attitudes were

to some extent conveyed through the manager or the working environment, they were also familiar to the employees prior to the terrorist incident and probably largely internalized from the past.

The most common factors that hindered the employees' capacity for work were either overlapping or closely related, regardless of whether they were categorized under the theme manager, social conditions, or work (Table S1). They were gathered under the final theme *high demands and lack of acceptance*; thus, the theme covered both management and the working environment in general. The subtheme *high demands* covered high work demands and little time for processing thoughts about the incident (moved from *manager*), as well as low degrees of flexibility, stressful work tasks, heavy work pressure, and unsuitable physical working conditions (moved from *work*). The subtheme *lack of acceptance for different reactions and individual needs* covered limited support, little acceptance of different reactions to the incident, low levels of empathy, lack of follow-up, and distinguishing between employees (moved from *manager*), as well as lack of community, lack of acceptance, differences in reactions and needs of employees, not being free to talk about the incident, and talking too much about the incident (moved from *social conditions*).

In the sixth step, we made the write-up of the results selecting text extracts that we considered representative of themes and sub-themes. We strived to build the quotes into an analytical narrative that illustrated the essence of the various themes.

Table S1. Intermediate stage in thematic analysis showing themes and relating factors that promoted and inhibited employees' capacity to work*.

Themes	Factors that increased capacity to work and helped to prevent sick leave	Factors that reduced capacity to work and increased the risk of sick leave
Manager	Support, recognition, acceptance, empathy, mastery beliefs, communication, low demands, facilitation, follow-up, encouragement of presence, competence in crisis and emergency preparedness	Limited support, lack of recognition, little acceptance of different reactions, lack of empathy, passive leadership, absent manager, high work demands, expectations of normal activity, little time for processing thoughts about the incident, lack of facilitation, lack of follow-up, poor communication, exclusion, distinguishing between employees, encouragement to take sick leave, lack of competence in crisis and emergency preparedness
Social conditions	Belonging, unity, community, colleague support, inclusion, recognition, being social, openness, good mood, humour, cooperation, volunteer spirit, expectations of continuing as before, drive, expectations of mutual support, talking about the incident, not talking too much about the incident	Lack of community, lack of colleague support, loneliness, lack of acceptance, sombre mood, conflicts, poor working environment, differences in reactions and needs of employees, not being free to talk about the incident, talking too much about the incident
Individual	Motivation, mastery beliefs, coping strategies, loyalty to the workplace, sense of duty, taking responsibility, accepting the situation, job commitment, job satisfaction, positive to work participation, setting boundaries, avoiding counterfactual thinking, robustness, health, family, leisure activities	Not taking responsibility for self, counterfactual thinking, vulnerability to stress, health, strains in the family
Work	Autonomy, flexibility, sense of meaningfulness, security, mastery, being useful, normalization, interesting work tasks, large workload, new work situation	Lack of flexibility, having little sense of meaning, stressful work tasks, high work demands, heavy work pressure, unsuitable physical working conditions, constant reminders about the incident, still being in the same vulnerable work situation, lack of safety measures, new work situation
Terrorist incident	Less exposed to the terrorist incident	Directly exposed to the terrorist event, proximity to the bomb, physically injured, being alone during the event

* Interviews held with 98 employees in Norwegian ministries after the 2011 Oslo bombing.
